# Monitoring the Dough Properties, Quality Characteristics and Volatile Compounds of Whole Wheat Bread Made by Different Sourdough Types during Frozen Storage

**DOI:** 10.3390/foods13091388

**Published:** 2024-04-30

**Authors:** Gorkem Ozulku

**Affiliations:** Department of Food Engineering, Faculty of Chemical and Metallurgical Engineering, Yildiz Technical University, İstanbul 34220, Türkiye; ozulkug@yildiz.edu.tr

**Keywords:** dough rheology, freezable water content, secondary structure, HS/GC-MS analyses

## Abstract

There is a need to increase the consumption of whole wheat bread (WWB) due to its health benefits by overcoming its poor technological quality and improving its sensory characteristics. In this study, sourdough bread-making and frozen dough technology were combined to provide fresh WWB at any time with better quality. Also, it was aimed to investigate the effects of three types of sourdough (type I, II, and IV) on the final quality of WWB during frozen storage (−30 °C, 14 and 28 days). The tan δ of WWB with type I sourdough was highest at the end of the frozen storage. Freezable water content was lower on day 0 for WWB with type II and IV sourdough than other bread types. No significant effect of frozen storage was observed in bread types in terms of an α helix structure, except for WWB with type I sourdough. A lower hardness increment was shown in WWB with baker’s yeast and WWB with type II sourdough over 14 days of frozen storage when compared to other bread types. WWB with type I sourdough and WWB with type IV sourdough were differentiated from other bread samples in volatile compound (VC) analysis on frozen storage days 28 and 0, respectively. The frozen storage of WWB with baker’s yeast and WWB with type II sourdough caused no notable changes in the VCs profile. These results suggest that a less detrimental effect of frozen storage was observed in WWB with type II sourdough, indicating a more favorable choice for producing WWB with sourdough.

## 1. Introduction

Whole wheat bread (WWB) is one of the representatives of whole grain foods that are strongly recommended to be consumed daily. From a nutritional perspective, WWB is a good source of polyphenols, dietary fibers, vitamins, and minerals. Regular consumption of such nutritional compounds has a role in reducing the risk of some diseases, such as cardiovascular diseases, cancer, diabetes, and obesity [1]. From an organoleptic perspective, WWB has a lower consumer acceptability when compared to refined wheat flour bread. A less specific volume, darker color, and denser texture are some drawbacks to WWB [2]. Therefore, there is a great interest in improving the organoleptic properties of WWB [3]. Several studies investigated the effect of some additives (emulsifiers, oxidizing agents) and enzymes (alpha-amylases, glucose oxidase, hemicellulases) to increase the quality characteristics of WWB [2,4].

Frozen dough technology is one of the bread-making processes and provides some advantages to the bakery industry in terms of production costs, the stability of product quality, and offering fresh bread to consumers at any time [5]. Despite these advantages, the frozen storage of bread doughs causes significant quality losses, such as lower specific volume and poor textural characteristics, similar to using whole wheat flour in bread making [6]. Many attempts have been conducted to decrease such quality problems, i.e., the modification of the frozen dough-making process, the increment of yeast freeze tolerance, and using some food additives [7]. Hydrocolloids to mitigate water mobility [8,9], anti-freeze additives to inhibit ice crystal growth [10,11], and emulsifiers as conventional frozen dough improvers [7] have been widely used in frozen dough technology. The addition of dietary fibers has also been investigated in many studies. Water-extractable arabinoxylan from rye bran showed an improvement by crystallizing less water during 60 days of frozen storage [12]. In a study by Adams et al. [13], wheat aleurone and bran were substituted with refined wheat flour in order to monitor the rheological properties and final bread quality of frozen dough. The results revealed that higher amounts of bound water and less freezable water were shown at −18 °C for 9 weeks, and higher bread volume was obtained from these flours than control flours (refined flour and whole wheat flour). Whole wheat flour was investigated by Bae et al. [14] in a frozen dough system, and WWB showed lower bread volume reduction and hardness increment when compared to the bread from refined flour after frozen storage (−20 °C for 4 weeks). In a recent study, the addition of enzymes and hydrocolloids into WWB made by frozen dough was investigated, and the optimal formulation was determined for WWB with xylanase (0.12 g/kg), lipase (0.25 g/kg) and xanthan gum (3.1 g/kg) [15].

Sourdough bread making can also be considered as a solution to overcome some quality disorders of WWB and other wholesome food. During sourdough fermentation, the formation of exopolysaccharides and the increase in water-extractable arabinoxylans can modify the gas-holding capacity of dough, leading to its beneficial effect on the loaf volume of wheat bread [3,16,17,18]. In whole wheat flour dough, cooperative fermentation by yeast and lactic acid bacteria (LAB) can provide stronger intermolecular interactions between gluten proteins and wheat starch by weakening the bran–protein interactions that have a detrimental effect on dough properties [1]. The microbial ecosystem of sourdough is dominated by yeast and lactic acid bacteria (LAB). Yeast in sourdough is mainly responsible for producing CO_2_ to leaven the bread, while LAB generates a variety of metabolites, i.e., exopolysaccharides and organic acids. This collaboration between LAB and yeast contributes to the texture, flavor, and shelf-life of WWB [1,17]. Different procedures have been applied to produce sourdoughs known as type I, type II, type III, and type IV. Type I sourdough production is characterized by traditional techniques, which include the daily refreshment of dough and spontaneous fermentation. Starter cultures have been used for faster, controllable, and large-scale sourdough production. This type of sourdough is called type II, which is convenient for industrial bakery. Type III sourdough is the powder form of type II and is usually used as an aroma carrier for bread-making. Type IV sourdough is prepared with the addition of starter cultures into mature sourdough in some artisanal bakeries and laboratory studies. It also has a similar production process as type I [19,20]. A few studies have been performed to investigate both the use of sourdough and frozen storage in WWB. The effect of sourdough on partially baked frozen WWB was studied by Novotni et al. [21], and it was reported that *Lactiplantibacillus plantarum* sourdough improved the quality attributes and retarded the firming rate of WWB.

Despite some studies that have been performed to increase the knowledge of WWB quality during frozen storage, there are some gaps with respect to the effects of sourdough types (type I, II, and IV) on WWB baked from frozen dough. The purpose of this study was to investigate the combined effects of sourdough bread-making and frozen dough technology on WWB to assess which sourdough type is more efficient for the frozen storage of WWB dough. Therefore, the dough properties and some quality characteristics of WWB were determined besides the volatile compounds of WWB during frozen storage (−30 °C, 14 and 28 days).

## 2. Materials and Methods

### 2.1. Materials

Refined flour (Sinangil, Eksim Co., İstanbul, Türkiye), whole wheat flour (Sinangil, Eksim Co., İstanbul, Türkiye), dry yeast (Pakmaya, İzmit, Türkiye), and salt (Billur, İzmir, Türkiye) were purchased from a local market. MRS (de Man, Rogosa, and Sharpe) agar, SDA (Sabouraud dextrose agar), MRS broth, Sabouraud dextrose broth (SDB), and peptone water were purchased from Merck (Darmstadt, Germany). Lactic acid bacteria (*Lacp. plantarum* LABE 29, *Levilactobacillus brevis* LABE 32) and *Saccharomyces cerevisiae* TGM 55 were obtained from the culture collection of Yildiz Technical University in the Department of Food Engineering. The isolation source of these bacteria and yeasts was wheat sourdough.

### 2.2. Sourdough Production

Refined flour (8.3% moisture, 1.04% fat, and 13.62% protein) was used for sourdough production. All sourdough types were prepared according to the protocols that are summarized in a study by Martin-Garcia et al. [20].

#### 2.2.1. Type I Sourdough Preparation

In total, 312.5 g of refined flour and 187.5 g of tap water were mixed in a dough mixer (KitchenAid, Benton Harbor, MI, USA) for 5 min to obtain sourdough with a 160 dough yield (DY, [(amount of flour + water)/(amount of flour) × 100]). Fermentation (Nuve TK 252, Ankara, Turkey) conditions were at 25 °C for 22 h. Daily propagation was performed by taking 10% of the previously fermented dough and mixing it with 281. A total of 25 g of flour and 168.75 g of tap water have a DY of 160. Fermentation is carried out under the same conditions (25 °C for 22 h). Sourdoughs are propagated daily for five days. The final pH of type I sourdough is 4.10.

#### 2.2.2. Type II Sourdough Preparation

*Lacp. plantarum* LABE 29 and *Levl. brevis* LABE 32 were cultivated on MRS agar and incubated at 37 °C for 48 h. After incubation, a single colony was picked from agar plates and subcultured twice in MRS broth. The cells were precipitated twice with sterile pure water by centrifugation (4500 rpm, 10 min). A similar procedure was performed for *S. cerevisiae* TGM 55 using SDA and SDB. The incubation conditions were at 30 °C for 48 h. 

Type II sourdough (DY 200) was prepared with refined flour (250 g), sterile tap water (205 g), and 45 mL of cell suspension, which consists of 15 mL of individual *S. cerevisiae* TGM 55 (final cell concentration in the dough of ca. 7.6 log cfu g^−1^) and 30 mL of LAB cell cultures with 8.3 log cfu g^−1^ of final cell concentration in the dough. After kneading all ingredients (KitchenAid, Benton Harbor, MI, USA) for 5 min, the dough was fermented at 30 °C for 22 h. The pH of type II sourdough was 3.72.

#### 2.2.3. Type IV Sourdough Preparation

Type II sourdough was produced again, and daily propagation was performed, as stated in the type I sourdough preparation to obtain type IV sourdough (DY~160). The pH of this sourdough was 3.91.

### 2.3. Whole Wheat Bread Making 

Whole wheat flour (10.09% moisture, 1.4% fat, and 16.67% protein) for bread making was used to produce whole wheat-yeasted bread (WWYB) and type I, type II, and type IV whole wheat sourdough bread (type I WWSB, type II WWSB, and type IV WWSB). WWYB was prepared with 60% tap water (*w*/*w* flour base (fb)), 2% dried baker’s yeast (*w*/*w*, fb), and 1.5% salt (*w*/*w*, fb). For WWSBs, 30% sourdough was added to the bread dough. All ingredients were blended with a dough mixer (KitchenAid, Benton Harbor, MI, USA) at speed 2 for 2 min and at speed 4 for 3 min for WWYB production. Sourdough and one part of water were mixed for 2 min before other ingredients were incorporated for WWSB production. The bread dough was divided into 165 g and shaped. After shaping, frozen storage was performed at −30 °C for 14 and 28 days. The fermentation process was carried out at 30 °C and 85% RH for 2 h (Nuve TK 252, Ankara, Turkey) for unfrozen dough samples and frozen dough samples after thawing (at 4 °C for 3 h). The bread doughs were baked at 235 °C for 25 min. (Maksan, Nevsehir, Turkey). Two replicates for each bread type were prepared.

### 2.4. Dynamic Rheological Measurements 

The unfrozen and frozen-thawed dough samples were subjected to a dynamic rheological analysis by a temperature-controlled rheometer (Antonpaar MCR 302, Graz, Austria). First, the dough sample (2 g) was placed on a parallel plate with a 2 mm gap. Then, an amplitude sweep test was performed to investigate the linear viscoelastic region, which was found to be 0.1%. The frequency sweep tests between 0.1 rad/s and 100 rad/s at 25 °C were conducted to determine the storage modulus (G′), loss modulus (G″), and tan δ values (G″/G′). Duplicate measurements were performed. The power law model was used to describe the model parameters, including intercepts (K′, K″) and slopes (n′, n″), according to the following Equations (1) and (2) [22]:

G′ = K′ (ω)n′(1)

G″ = K″ (ω)n″(2)

### 2.5. Determination of Freezable Water Content 

In total, 10 mg of the dough sample was weighed in an aluminum tray and placed in DSC (TA instrument Q20, New Castle, DE, USA) with an empty aluminum tray as the reference. The cooling of the sample was performed first from 20 °C to −40 °C at a rate of 5 °C/min and kept isothermal for 5 min. The heating stage was carried out from −40 °C to 10 °C at a rate of 5 °C/min. [23]. The enthalpy (J/g) of the melting (∆H) was recorded, and freezable water (FW) content was determined using the following Equation (3):

FW(%) = ∆H/∆Ho × Wc × 100(3)

∆H: the enthalpy (J/g) of the melting peak of the endothermic curve;
∆Ho: the enthalpy (334 J/g) of the melting peak of pure water;
Wc: the total water content (%) of the dough.

A rapid moisture analyzer (Radwag MR50, Poznan, Poland) was used to measure the total water content. The analysis was carried out with 2 replications for each sample.

### 2.6. Fourier Transform Infrared Spectroscopy (FT-IR)

The FT-IR spectra of lyophilized dough samples were recorded at a resolution of 2 cm^−1^ with wave numbers from 400 to 4000 cm^−1^ during 16 scans per spectra by the FTIR spectrometer equipped with a DLa TGS detector (Bruker Tensor 27, Bremen, Germany). Originlab software (version 9.0 PRO, OriginLab Corporation, Northampton, MA, USA) was used for the interpretation of changes in the overlapping amide I band (1600–1700 cm^−1^) by deconvolution [24]. Three measurements were conducted for each sample. 

### 2.7. Determination of Quality Characteristics of Breads

#### 2.7.1. Specific Volume 

The rapeseed displacement method was used to measure the volume of bread [25]. The specific volume was determined by dividing the volume (mL) of the bread sample by its weight (g). 

#### 2.7.2. Texture Profile Analysis (TPA)

Textural properties of crumbs (hardness, springiness, cohesiveness, and chewiness) were measured by the texture analyzer (SMS TA.XT2 Plus, Glasgow, UK) using a 5 kg load cell and a 36 mm diameter cylindrical compression probe. Firstly, each bread was sliced to 12.5 mm in thickness, and a cylindrical crumb was taken from the center of each slice. Two cylindrical crumbs (height: 25 mm, diameter: 36 mm) were subjected to the texture analyses. The parameters of TPA were as follows: 50% compression, 5.0 mm/s test speed, and 5 s delay time between the two compression cycles. Triplicate measurements were performed for each bread type.

#### 2.7.3. Color Analysis 

The lightness (L*), redness (a*), and yellowness (b*) values of the bread crusts and crumbs were determined using a Chromameter (CR-100 Konica Minolta, Tokyo, Japan). Six measurements were taken from each sample. 

### 2.8. Determination of Volatile Compounds of Breads

The volatile compounds of bread were determined by HS/GC-MS analysis using a GCMS-QP2010 (Shimadzu, Milan, Italy) combined with a CTC-Combi-PAL autosampler (Bender and Hobein, Zurich, Switzerland). The breadcrumb (~3 g) was transferred into 20 mL of headspace vials agitated for 15 min at 70 °C by the autosampler system. Chromatographic separation was performed using helium as a carrier gas (1.71 mL/min) and a Restec (Bellefonte, PA, USA) Rtx-5MS fused silica capillary column (30 m × 0.25 mm (internal diameter) at 0.25 μm. The parameters of the autosampler were adjusted according to the method of Tulukcu et al. [26]. The temperature program of gradient analysis was as follows: 40 °C (3 min); 40–176 °C (8 °C/min); and 176 °C (20 min). The temperature of the injector, the pressure value, and the linear velocity were 150 °C, 95.8 kPa, and 47.1 cm/s, respectively [27]. The identification of the detected volatile compounds was carried out by the commercial mass spectra libraries (NIST27 and WILEY7).

### 2.9. Statistical Analysis 

The results (mean ± standard deviation) were assessed using analysis of variance (ANOVA). One-way ANOVA followed by Duncan’s post hoc test was performed with two approaches (SPSS 19.0 software, Chicago, IL, USA). The single fixed factor was chosen as the bread type, and the results were evaluated according to the storage days in the first approach. The second approach was to fix the storage days and evaluate the results according to the bread types. *p* < 0.05 was considered significant. Principal component analysis (PCA) was performed to discriminate the volatile compounds of bread samples by JMP Pro (Ver 17, SAS Inc., Cary, NC, USA). The percentage composition (area %) of identified volatile compounds by HS/GC-MS analysis was used in PCA.

## 3. Results and Discussion

### 3.1. Rheological Properties 

The parameters (K′, n′, K″, and n″) of the power-law model of WWYB and WWSB doughs during frozen storage are shown in Table 1. The range of R2 values of the power-law equation is between 0.93 and 0.99 for G′ and 0.97 and 0.99 for G″. The solid character index (K′) was higher than the liquid character index (K″) for all samples and storage times, indicating solid-like behavior [28]. No significant differences were observed between the bread dough samples for consistency coefficients (K′, K″) on day 0 of frozen storage, except for type II WWSB dough. Type II WWSB dough exhibited a lower K′ and K″ value, showing less elastic and viscous dough [29]. Frozen storage led to a reduction in the K′ and K″ values of type II WWSB on days 14 and 28 and type IV WWSB on day 28. This could be due to the acidic characteristics of these sourdough types. In the present study, type IV (pH 3.91) and type II (pH 3.72) sourdough had higher acidity than type I sourdough (pH 4.10). Both acidic conditions and frozen storage resulted in a weaker protein network structure [30,31] and, in turn, a decrease in dough elasticity and viscosity. The flow behavior index (n′, n″) of fresh dough samples was between 0.17 and 0.25. Jin et al. [32] reported the n′ and n″ values for wheat dough containing wheat bran (10–30%) as between 0.17 and 0.22. 

**Figure 1:**
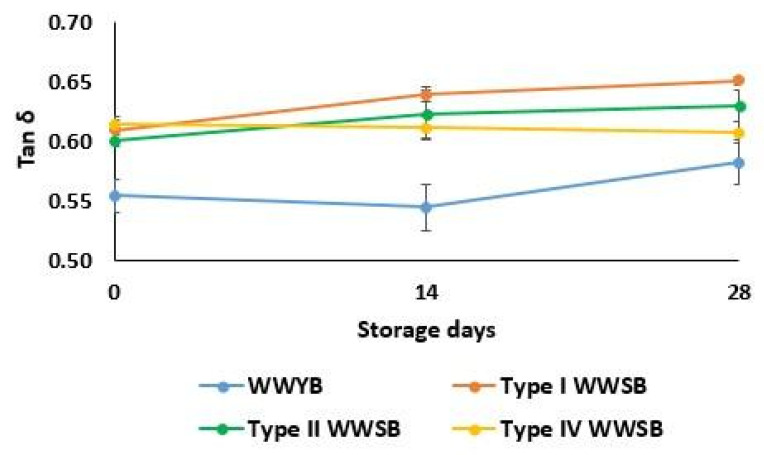
Tan δ value (at 1 Hz) of dough samples. WWYB: Whole wheat yeasted bread; WWSB: whole wheat sourdough bread. Types I, II, and IV are sourdough types.

The tan δ value (G″/G′) at 1 Hz is presented in Figure 1. The tan δ values for all dough samples were lower than one, which reflects the predominance of elastic characteristics [33]. The tan δ values of WWSB doughs were higher than WWYB on day 0 (Figure 1). The lower tan δ value indicates polymerization in the dough system [31]. Therefore, WWSB doughs showed lower polymer content due to having higher tan δ values. This could be attributed to the hydrolysis of gluten proteins under acidic conditions [31]. Type I WWSB dough showed an increase in its tan δ value after 14 days of storage and had the highest tan δ value in all at the end of the storage period (Figure 1). This different behavior of type I WWSB from other WWSB dough types in terms of the tan δ value may be explained by the fact that the damaging effect of frozen storage on the gluten matrix is higher than that of other WWSB doughs. Another possible explanation for this result could be that sourdough types with higher acidity lead to the formation of non-starch polysaccharides, which bind available water in the dough system during fermentation [17,34]. Type I sourdough had a lower acidity (pH 4.10), and, in turn, the hydrolysis effect of sourdough fermentation and formation of non-starch polysaccharides were limited when compared to other sourdough types.

### 3.2. Freezable Water (FW) Content of Frozen Bread Dough

**Figure 2:**
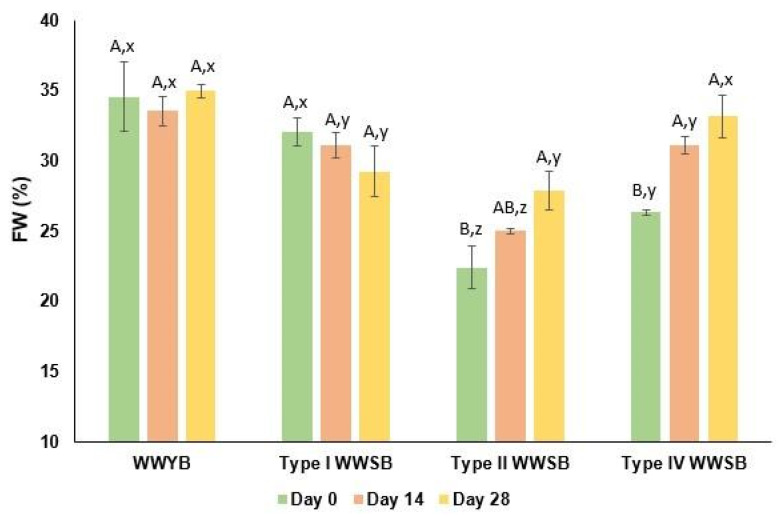
Freezable water (FW) content of dough samples. Different uppercase letters show significant differences during frozen storage of the same bread type (*p* < 0.05). Different lowercase letters show significant differences between bread types for the same frozen storage time (*p* < 0.05). WWYB: Whole wheat yeasted bread; WWSB: whole wheat sourdough bread. Types I, II, and IV are sourdough types.

The FW contents of dough samples are illustrated in Figure 2. On day 0 of frozen storage, type II WWSB showed the lowest FW content, followed by type IV WWSB, while no significant differences were observed between WWYB and type I WWSB. The FW content indicates the water content available to form ice crystals during frozen storage, and this content can be reduced by the addition of certain ingredients with water-binding potential [35]. In this study, type II and IV WWSB had a lower FW content than WWYB and type I WWSB on day 0 (Figure 2). This could be due to the effect of type II and type IV sourdough fermentation on the macromolecules of whole wheat dough. The fermentation of bread with type II and IV sourdough can lead to the production of some components that interact with water, such as non-starch polysaccharides, due to the hydrolytic effect of sourdough acidity [17,36]. After 14 days of frozen storage, the FW content of type IV WWSB increased (*p* < 0.05) and showed no significant differences for the rest of the storage period. For type II WWSB, a notable rise in FW content was observed on the 28th day of frozen storage (*p* < 0.05). The increase in FW content during frozen storage was the result of the conversion of bound water to freezable water [35]. Frozen storage caused no effect on the FW content of WWYB and type I WWSB. In a study by Adams et al. [13], a significant increment in the FW content of whole wheat flour dough was not shown for over 3 weeks of frozen storage (−18 °C). This result is in line with the results from the present study in terms of the FW content of WWYB during frozen storage.

### 3.3. Secondary Structures of Proteins in Bread Doughs

FT-IR spectroscopy was used to observe the effect of frozen storage on the secondary structures of protein. The amide I (1700–1600 cm^−1^) in FTIR, which is the main characteristic band of proteins, includes α-helix, β-sheet, β-turn, and random coil conformations. While α-helix and β-sheet conformations are relatively ordered and support the skeletal structure of the protein, the random coil structure is the most disordered and weak structure in the protein [37]. The results for all bread doughs are presented in Table 2. The major secondary structures showed differences between bread types. Additionally, the same bread type displayed different major secondary structures during frozen storage (Table 2). The major structure during frozen storage included antiparallel β sheets for WWYB and type I WWSB. For type II WWSB, there were random coils on day 0, α helices on day 14, and antiparallel β sheets on day 28. The dominant structure of type IV WWSB included α helices on day 0, antiparallel β sheets on day 14, and β turns on day 28. Bread types and frozen storage had no significant effect on the β sheets structure. The β turns structure decreased significantly on the 28th day of frozen storage for WWYB. As for the α helices, which were considered relatively orderly and stable in structure, their increasing effect was shown on the 28th day of frozen storage for type I WWSB. No significant changes were observed for other bread types during frozen storage in terms of α helices. Type I WWSB also had a lower α-helix structure in all bread types on days 0 and 14. The effect of frozen storage on the α-helix structure was reported contradictorily for wheat flour dough. According to Zhou et al. [35], the α-helix structure a showed downward trend during 28 days of frozen storage (−18 °C). On the other hand, the increment of α helices was reported after 60 days of frozen storage (−18 °C) in a study by Yu et al. [38]. Bock et al. [39] indicated that increasing the bran content from 0 to 10% in wheat flour dough caused a reduction in α-helix and β-turn structures and an increment in β-sheet and random structures. In the present study, whole wheat flour was used, and this could potentially account for the different results when compared to other studies. Also, sourdough of different types was incorporated into bread making, and *Lacp. plantarum*, which is frequently isolated in sourdough, was used for type II sourdough production in this study. Wheat sourdough fermentation with *Lacp. plantarum* resulted in an increase in the β-turns and a decrease in the α-helix structure of the glutenin macropolymer, according to the study by Wang et al. [40] and Yin et al. [41]. Similar results in terms of decreasing the α-helix structure were also obtained in this study when compared to WWYB with type I and II WWSB on day 0 (Table 2). No significant effect of frozen storage on the secondary structure of WWYB dough was observed except for the conformation of β turns. Also, some rheological properties (K′ and K″) and the FW content of WWYB dough did not change significantly during frozen storage, as shown in Table 1 and Figure 2, indicating that WWYB dough maintained its stability during frozen storage. 

### 3.4. Quality Characteristics of Bread

#### 3.4.1. Specific Volume and Texture

**Figure 3:**
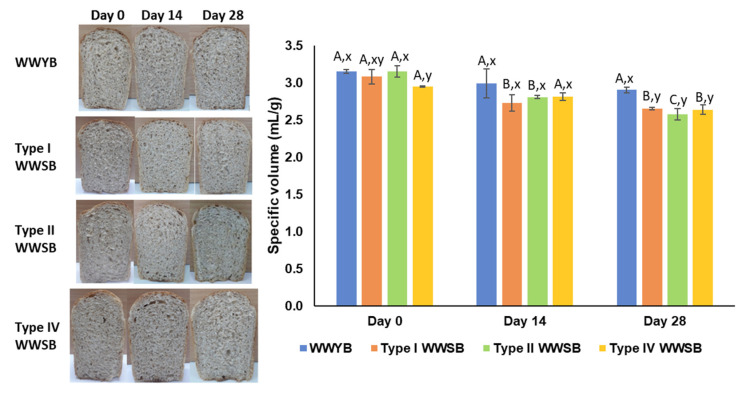
Specific volume and images of bread samples. Different uppercase letters show significant differences during frozen storage of the same bread type (*p* < 0.05). Different lowercase letters show significant differences between bread types for the same frozen storage time (*p* < 0.05). WWYB: Whole wheat yeasted bread; WWSB: whole wheat sourdough bread. Types I, II, and IV are sourdough types.

The specific volume (SV) and textural properties of bread samples are exhibited in Figure 3 and Table 3, respectively. No significant differences were observed in terms of SV and hardness (N) or the value of fresh bread samples (day 0). The improvement in the sourdough microorganism on the quality characteristics of WWB has been reported in some studies [18,42,43]. However, in the study by Demirkesen-Bicak et al. [44], an SV reduction and hardening effect were observed in WWBs made by sourdough. Karaman et al. [45] obtained the lowest hardness value for WWBs made by sourdough containing a phytase-active yeast combination (*Saccharomyces cerevisiae* + *Pichia membranifaciens*). Yildirim and Arici [46] and Shen et al. [42] found higher volume and lower hardness values using the combination of yeast and LAB. Sourdough types, bread formulation, process parameters, and strain selection can cause such differences in the quality of WWB. Verdonck et al. [3] investigated the effect of process parameters in whole meal wheat bread made with sourdough and baker’s yeast and indicated that process optimization is important for studying the quality aspects of WWB. The results from the present study are in line with the study by Verdonck et al. [3] since no significant differences between WWB with type I sourdough and yeasted WWB in terms of SV were found (Figure 3). The effect of frozen storage was remarkable for WWSBs in terms of SV and textural properties (Figure 3 and Table 3). A significant reduction in SV was observed for type I WWSB on storage day 14 and for type IV WWSB on storage day 28, while a gradual decrease was shown for type II WWSB during frozen storage. No significant effect was detected for the SV of WWYB during frozen storage (*p* > 0.05). Also, SV loss was more distinct for type II WWSB (18.1%) for 28 days of storage, while WWYB showed a lower SV reduction (7.9%). A similar value for volume reduction was observed in the study of Bae et al. [14] for whole wheat bread during 4 weeks of frozen storage.

The textural properties of WWYB were affected by frozen storage. The hardness value (N) of WWYB increased significantly on day 28 (*p* < 0.05), and a 38.3% increment was observed. Different results were reported in relation to the quality of WWB during frozen storage. In a study by Adams et al. [13], whole wheat flour bread showed an increase in the loaf volume for up to 3 weeks of frozen storage (−18 °C), and a significant reduction was observed after 3 weeks. It was also reported that the crumb firmness of WWB did not change significantly during this period [13]. He et al. [15] observed a notable reduction in SV and an increment in the hardness of WWB baked from frozen dough after 21 days of storage. The hardness value of type I and IV WWSBs increased on storage day 14 and remained stable on day 28, while a gradual increment was shown for type II WWSBs. However, the increment level of hardness was 31.9% for type II WWSB on the 14th day of storage, which is lower than other WWB types with sourdough. The springiness value in the textural evaluation of bread is an important parameter that reveals the degree of recovery after removing the compressing force [47]. The springiness value of bread samples on day 0 varied from 0.88 to 0.93, and the highest springiness value was shown for types I and IV WWSB (Table 3). Begum et al. [48] stated that lower hardness and higher springiness values were the indicators of improved textural properties of bread. From this point of view, sourdough bread making (type I and IV) enhanced the bread’s textural parameters in terms of the springiness value when compared to WWYB. After frozen storage (−30 °C, 28 days), a significant reduction was observed for all bread types, ranging between 0.76 and 0.84 (Table 3). A decrease in the springiness was also attributed to quality loss in bread, according to Silvas-García et al. [47]. Cohesiveness, which shows the strength of internal linkages in the samples, was higher in WWSBs than in WWYBs. Indeed, 28 days of frozen storage caused a decreasing effect on this value, similar to the springiness value (Table 3). This also indicated the disruption of gluten strength, causing quality losses in the bread. The chewiness value of the bread samples showed no significant differences on day 0. This result is also in line with the hardness result of the bread samples since the chewiness value is directly proportional to hardness. After 28 days of frozen storage, the chewiness value of type II WWSB was the highest (*p* < 0.05). This shows that higher energy is required to masticate it [49].

#### 3.4.2. Color

Bread color is considered as important physical criteria that influences consumer preferences and is strongly affected by formulations and the baking process [17]. Table 4 shows the crust and crumb characteristics of bread samples. WWSBs had a higher L* value than WWYB on day 0 of frozen storage. In this study, the use of sourdough prepared with refined wheat flour possibly caused the bleaching effect on whole wheat bread and, in turn, increased the L* value. Frozen storage resulted in an increment of the crust L* value in WWYB on day 14 and remained stable for the rest of the storage time. A similar observation was reported by He et al. [15] for whole wheat control bread during 21 days of frozen storage, identifying a less intense Maillard reaction in bread crusts. The possible explanation for this result was that less yeast activity occurred due to frozen storage, causing a lower supply of substrates for the Maillard reaction [31]. No significant effect on the crust L* value in WWSBs was observed due to frozen storage except for type IV WWSB. Frozen storage caused some variations in the crust b* value of type II and IV WWSBs, while a noticeable effect was shown for type I WWSB and WWYB. WWSBs exhibited a higher crumb L* value than WWYB. Frozen storage caused a reduction in the crumb L* value of type IV WWSB, while no significant effect was shown for type I and II WWSBs. At the end of 28 days of frozen storage, no effect was shown on the crumb b* values of the samples (Table 4).

### 3.5. Volatile Compounds

**Figure 4:**
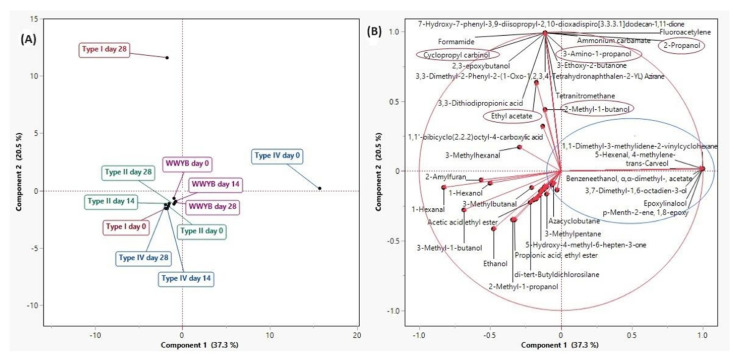
PCA plots for the discrimination of bread samples (**A**) and volatile compounds (**B**) WWYB: Whole wheat yeasted bread; WWSB: whole wheat sourdough bread. Types I, II, and IV are sourdough types.

The interactions of different bread types and frozen storage times with volatile compounds (VCs) of bread samples were analyzed by PCA. The first two components, component 1 (37.3%) and component 2 (20.5%), explained 57.8% of the total variance (Figure 4). Type I WWSB on day 28 and type IV WWSB on day 0 were differentiated from other bread samples, as seen in Figure 4A. The VCs are mainly located on the left side of the chart (Figure 4B). The bread samples on the negative side of component 1 had more VCs when compared to type IV WWSB on day 0. The VCs, such as 5-hexenal-4-methylene-, trans-carveol, epoxylinalool, 3,7-dimethyl-1,6-octadien-3-ol, and p-menth-2-ene-1,8-epoxy, showed a close correlation with type IV WWSB on day 0 (Figure 4B). The VCs, such as 2-propanol, 3-amino-1-propanol, 2-methyl-1-butanol, and cyclopropyl carbinol, located on the positive side of component 2, were closely connected to type I WWSB on day 28. Among these VCs, cyclopropyl carbinol was reported in brown fermented milk as a flavor component [50], and 2-methyl-1-butanol was one of the products of yeast metabolism [51]. Ethyl acetate, which is characterized as having a fruity odor, was also more highly associated with type I WWSB on day 28 (Figure 4B). Some alcohols (ethanol, 3-methyl-1-butanol, 2-methyl-1-propanol, and 1-hexanol), some aldehydes (1-hexanal, 3-methylbutanal, 3-methylhexanal), a ketone (5-Hydroxy-4-methyl-6-hepten-3-one), an alkane (3-methyl pentane), a furan (2-amyl furan (2-pentylfuran)), and an ester (propionic acid, ethyl ester (ethyl propionate)) were detected in most of the bread samples and located on the negative sides of both component 1 and 2. As for the aroma characteristics of these compounds, 2-methyl-1-propanol and 3-methyl-1-butanol were described to have an alcoholic odor. 3-methyl-1-butanol is produced from leucine by yeast metabolism via the Ehrlich pathway, while 1-hexanol is the product of reduction reactions of aldehydes with alcohol dehydrogenases [52,53]. Hexanal, which is an indicator of lipid oxidation, is related to a fishy and grassy odor [51]. 2-amyl furan (2-pentylfuran) is described as the most aroma-active component in breadcrumbs with a floral and fruity odor [52,53]. The frozen storage of bread dough samples caused no notable changes in the profile of VCs, especially WWYB and type II WWSB samples since they were classified on the negative sides of components 1 and 2 (Figure 4A).

## 4. Conclusions

Whole wheat bread has a variety of health benefits, but it has poor technological quality. Extensive research has been performed to improve its quality using sourdough technology. However, revealing the effects of frozen storage on WWB quality is important since frozen storage has some advantages for the bakery industry. Therefore, this study was designed to expand the knowledge of WWB making with the combination of frozen dough and sourdough technology. According to the results, sourdough types and the duration of frozen storage are important for WWB. The crumb hardness of WWB with types II and IV sourdough was lower than WWB with baker’s yeast, but these differences were not statistically significant. Frozen storage caused bread volume reduction and an increment in hardness in WWBs with sourdough, and a significant effect was shown on the 14th day of frozen storage. No significant differences were shown between WWB with baker’s yeast and type II sourdough in terms of crumb hardness on the 14th day of frozen storage. Also, frozen storage did not change the profile of their volatile compounds. The results of the present study suggest that WWB with type II sourdough is more promising for 14 days of frozen storage as it has lower freezable water content, lower hardness increment, and a stable volatile compound profile when compared to other sourdough types. Type II sourdough is an industrial type of sourdough that requires minimum time for production. This can be a practical impact of this study for the bakery industry. More improvement can be obtained in further research by optimizing the fermentation process and dough formulation with other food additives.

## Figures and Tables

**Table 1:** Dynamic rheological properties (constants of power low equation) of dough samples.

		G′ = K′(ω)^n′^			G″ = K″(ω)^n″^		
Bread Type	Storage Time (Day)	K′ (×10^3^ Pa)	n′	R^2^	K″ (×10^3^ Pa)	n″	R^2^
WWYB	0	8.39 ± 0.71 ^Ax^	0.23 ± 0.01 ^Ax^	0.96	5.86 ± 0.68 ^Ax^	0.17 ± 0.01 ^By^	0.98
	14	8.15 ± 0.29 ^Ax^	0.21 ± 0.00 ^ABy^	0.97	4.98 ± 0.06 ^Ax^	0.20 ± 0.00 ^Az^	0.99
	28	8.66 ± 1.13 ^Ax^	0.21 ± 0.01 ^Bz^	0.98	5.58 ± 0.73 ^Ax^	0.19 ± 0.00 ^Az^	0.98
Type I WWSB	0	8.35 ± 0.12 ^Ax^	0.25 ± 0.01 ^Bx^	0.93	5.77 ± 0.01 ^Ax^	0.23 ± 0.01 ^Cx^	0.97
	14	7.38 ± 0.63 ^Ax^	0.27 ± 0.01 ^Bx^	0.97	4.83 ± 0.25 ^Ax^	0.28 ± 0.00 ^Bx^	0.99
	28	9.36 ± 1.60 ^Ax^	0.34 ± 0.00 ^Ax^	0.96	6.10 ± 1.02 ^Ax^	0.35 ± 0.00 ^Ax^	0.98
Type II WWSB	0	4.98 ± 0.02 ^Ay^	0.25 ± 0.00 ^Ax^	0.97	3.44 ± 0.03 ^Ay^	0.23 ± 0.00 ^Bx^	0.99
	14	4.09 ± 0.07 ^Cy^	0.26 ± 0.02 ^Ax^	0.99	2.51 ± 0.01 ^Cy^	0.28 ± 0.01 ^Ax^	0.99
	28	4.54 ± 0.05 ^By^	0.23 ± 0.00 ^Ay^	0.99	2.74 ± 0.11 ^By^	0.26 ± 0.01 ^ABy^	0.99
Type IV WWSB	0	7.50 ± 0.39 ^Ax^	0.25 ± 0.01 ^Ax^	0.95	5.12 ± 0.20 ^Ax^	0.23 ± 0.01 ^Bx^	0.99
	14	8.07 ± 0.62 ^Ax^	0.25 ± 0.00 ^Ax^	0.97	5.28 ± 0.21 ^Ax^	0.26 ± 0.00 ^Ay^	0.99
	28	5.44 ± 0.37 ^By^	0.23 ± 0.01 ^Byz^	0.98	3.56 ± 0.12 ^By^	0.24 ± 0.01 ^ABy^	0.99

Different uppercase letters show significant differences during the frozen storage of same bread type (*p* < 0.05). Different lowercase letters show significant differences between bread types for the same frozen storage time (*p* < 0.05). WWYB: Whole wheat yeasted bread; WWSB: Whole wheat sourdough bread. Types I, II and IV are sourdough types.

**Table 2:** The secondary structures of dough samples.

	Bread Type				
	Storage Time (Day)	WWYB	Type I WWSB	Type II WWSB	Type IV WWSB
β-sheets (1682–1696 cm^−1^)	0	5.87 ± 1.92 ^Ax^	4.24 ± 0.97 ^Ax^	8.83 ± 5.05 ^Ax^	7.42 ± 1.88 ^Ax^
	14	5.61 ± 2.77 ^Ax^	8.25 ± 3.83 ^Ax^	8.82 ± 6.20 ^Ax^	9.89 ± 0.01 ^Ax^
	28	8.88 ± 0.63 ^Ax^	8.85 ± 4.20 ^Ax^	6.75 ±3.11 ^Ax^	8.76 ± 4.54 ^Ax^
β-turns (1662–1681 cm^−1^)	0	24.16 ± 2.59 ^Ax^	20.91± 3.53 ^Ax^	21.22 ± 1.89 ^Ax^	20.08 ± 1.55 ^Ax^
	14	22.18 ± 1.30 ^ABx^	21.77 ± 2.59 ^Ax^	17.89 ± 4.56 ^Ax^	15.52 ± 0.21 ^Ax^
	28	18.03 ± 1.15 ^Bx^	15.26 ± 0.07 ^Ax^	18.59 ± 2.33 ^Ax^	22.60 ± 4.88 ^Ax^
α-helices (1650–1660 cm^−1^)	0	23.56 ± 1.36 ^Ax^	13.44 ± 2.69 ^By^	16.15 ± 5.29 ^Axy^	22.86 ± 2.58 ^Ax^
	14	24.86 ± 4.38 ^Ax^	12.36 ± 1.10 ^By^	24.76 ± 2.23 ^Ax^	22.99 ± 2.23 ^Ax^
	28	21.70 ± 0.07 ^Axy^	25.61 ± 4.30 ^Ax^	21.60 ± 2.36 ^Axy^	16.90 ± 2.41 ^Ay^
Random coils (1640–1650 cm^−1^)	0	19.93 ± 5.44 ^Ax^	16.86 ± 0.40 ^Ax^	21.40 ± 1.09 ^Ax^	22.82 ± 4.37 ^Ax^
	14	16.43 ± 1.73 ^Axy^	13.98 ± 1.00 ^By^	17.12 ± 2.65 ^Axy^	20.56 ± 0.41 ^Ax^
	28	20.44 ± 1.00 ^Ax^	19.43 ± 0.93 ^Ax^	21.97 ± 2.07 ^Ax^	20.38 ± 3.40 ^Ax^
Antiparallel β-sheets (1615–1637 cm^−1^)	0	24.27 ± 3.26 ^Ay^	40.72 ± 0.50 ^Ax^	21.36 ± 4.03 ^Ay^	20.46 ± 0.39 ^Ay^
	14	27.95 ± 2.35 ^Ay^	37.97 ± 2.15 ^Ax^	23.62 ± 1.18 ^Ay^	23.93 ± 3.68 ^Ay^
	28	28.39 ± 0.16 ^Ax^	27.44 ± 1.43 ^Bx^	23.30 ± 4.52 ^Axy^	18.83 ± 1.53 ^Ay^
Intermolecular β-sheets (1612–1614 cm^−1^)	0	2.22 ± 0.97 ^Az^	3.83 ± 0.78 ^Ay^	11.03 ± 1.29 ^Ax^	6.37 ± 1.85 ^By^
	14	2.97 ± 0.30 ^Ay^	5.66 ± 1.18 ^Axy^	7.79 ± 2.07 ^Ax^	7.12 ± 2.06 ^Bxy^
	28	2.56 ± 1.01 ^Ay^	3.40 ± 0.46 ^Ay^	7.79 ± 3.46 ^Axy^	12.53 ± 0.89 ^Ax^

Different uppercase letters show significant differences during frozen storage for the same bread type (*p* < 0.05). Different lowercase letters show significant differences between bread types for the same frozen storage time (*p* < 0.05). WWYB: Whole wheat yeasted bread; WWSB: whole wheat sourdough bread. Types I, II, and IV are sourdough types.

**Table 3:** Effects of frozen storage and bread types on the textural properties of breads.

	Bread Type				
	Storage Time (Day)	WWYB	Type I WWSB	Type II WWSB	Type IV WWSB
Hardness (N)	0	3.84 ± 0.13 ^Bx^	3.91 ± 0.25 ^Bx^	3.63 ± 0.11 ^Cx^	3.41 ± 0.69 ^Bx^
	14	4.35 ± 0.42 ^By^	5.78 ± 0.30 ^Ax^	4.79 ± 0.66 ^By^	6.26 ± 0.76 ^Ax^
	28	5.31 ± 0.55 ^Az^	6.14 ± 0.77 ^Ayz^	7.81 ± 0.37 ^Ax^	6.63 ± 0.54 ^Ay^
Springiness	0	0.87 ± 0.03 ^ABz^	0.92 ± 0.02 ^Axy^	0.88 ± 0.01 ^Ayz^	0.93 ± 0.02 ^Ax^
	14	0.94 ± 0.05 ^Ax^	0.83 ± 0.03 ^By^	0.85 ± 0.01 ^By^	0.83 ± 0.01 ^By^
	28	0.79 ± 0.08 ^Bx^	0.84 ± 0.01 ^Bx^	0.81 ± 0.01 ^Cx^	0.76 ± 0.05 ^Bx^
Cohesiveness	0	0.75 ± 0.02 ^By^	0.79 ± 0.01 ^Ax^	0.79 ± 0.02 ^Ax^	0.80 ± 0.01 ^Ax^
	14	0.80 ± 0.01 ^Ax^	0.75 ± 0.01 ^By^	0.76 ± 0.01 ^By^	0.75 ± 0.01 ^By^
	28	0.75 ± 0.02 ^Bx^	0.75 ± 0.01 ^Bx^	0.75 ± 0.01 ^Bx^	0.74 ± 0.02 ^Bx^
Chewiness (N)	0	2.53 ± 0.21 ^Bx^	2.82 ± 0.21 ^Bx^	2.52 ± 0.12 ^Cx^	2.53 ± 0.50 ^Bx^
	14	3.27 ± 0.15 ^Ayz^	3.61 ± 0.15 ^Axy^	3.10 ± 0.42 ^Bz^	3.87 ± 0.39 ^Ax^
	28	3.14 ± 0.40 ^Az^	3.86 ± 0.47 ^Ayz^	4.76 ± 0.23 ^Ax^	3.72 ± 0.43 ^Ayz^

Different uppercase letters show significant differences during frozen storage of the same bread type (*p* < 0.05). Different lowercase letters show significant differences between bread types for the same frozen storage time (*p* < 0.05). WWYB: Whole wheat yeasted bread; WWSB: whole wheat sourdough bread. Types I, II, and IV are sourdough types.

**Table 4:** Color properties of breads.

		Crust			Crumb		
Bread Type	Storage Time (Days)	L*	a*	b*	L*	a*	b*
WWYB	0	48.63 ± 2.25 ^By^	12.01 ± 0.68 ^Ax^	19.71 ± 1.75 ^Bz^	63.61 ± 1.08 ^By^	3.91 ± 0.27 ^Bx^	17.38 ± 0.33 ^Ax^
	14	53.84 ± 2.76 ^Ax^	11.43 ± 0.42 ^Axy^	20.49 ± 1.94 ^Bx^	68.97 ± 1.00 ^Ax^	3.69 ± 0.27 ^Bx^	17.72 ± 0.93 ^Ax^
	28	53.35 ± 2.05 ^Ax^	10.97 ± 0.45 ^By^	22.97 ± 1.02 ^Ax^	63.73 ± 0.99 ^By^	4.33 ± 0.26 ^Ax^	17.82 ± 0.42 ^Ax^
Type I WWSB	0	55.65 ± 2.48 ^Ax^	10.65 ± 0.79 ^By^	23.66 ± 0.96 ^Ax^	64.83 ± 3.15 ^Axy^	3.09 ± 0.26 ^By^	16.86 ± 0.91 ^ABx^
	14	53.60 ± 4.77 ^Ax^	11.77 ± 1.58 ^ABx^	22.82 ± 1.95 ^Ax^	64.13 ± 0.85 ^Az^	3.21 ± 0.30 ^ABy^	16.36 ± 0.41 ^Bz^
	28	52.47 ± 2.92 ^Ax^	12.47 ± 0.78 ^Ax^	19.95 ± 1.95 ^By^	64.73 ± 0.80 ^Axy^	3.53 ± 0.26 ^Ay^	17.42 ± 0.32 ^Axy^
Type II WWSB	0	52.84 ± 2.88 ^Ax^	11.07 ± 0.99 ^Axy^	21.54 ± 1.44 ^Ay^	67.06 ± 1.36 ^Ax^	3.30 ± 0.32 ^Ay^	16.87 ± 0.56 ^Ax^
	14	52.09 ± 2.46 ^Ax^	11.48 ± 0.37 ^Ax^	17.79 ± 1.99 ^By^	66.03 ± 0.84 ^Ay^	3.33 ± 0.25 ^Axy^	17.15 ± 0.39 ^Axy^
	28	53.51 ± 4.07 ^Ax^	11.42 ± 0.98 ^Ax^	20.77 ± 2.41 ^Ay^	65.77 ± 1.10 ^Ax^	3.33 ± 0.14 ^Ay^	17.26 ± 0.28 ^Ay^
Type IV WWSB	0	53.93 ± 3.60 ^Ax^	10.98 ± 1.33 ^Bxy^	23.52 ± 1.64 ^Ax^	66.53 ± 1.60 ^Ax^	3.09 ± 0.35 ^By^	16.80 ± 0.52 ^Ax^
	14	49.67 ± 2.57 ^Bx^	12.49 ± 0.45 ^Ax^	20.74 ± 2.13 ^Bx^	65.36 ± 0.58 ^ABy^	3.68 ± 0.20 ^Ax^	16.62 ± 0.40 ^Ayz^
	28	50.62 ± 1.99 ^ABx^	12.31 ± 0.59 ^Ax^	22.94 ± 1.01 ^Ax^	64.34 ± 1.08 ^By^	3.59 ± 0.38 ^Ay^	16.98 ± 0.56 ^Ayz^

Different uppercase letters show significant differences during frozen storage of the same bread type (*p* < 0.05). Different lowercase letters show significant differences between bread types for the same frozen storage time (*p* < 0.05). WWYB: Whole wheat yeasted bread; WWSB: whole wheat sourdough bread. Types I, II, and IV are sourdough types.

## Data Availability

The original contributions presented in the study are included in the article, further inquiries can be directed to the corresponding author.
